# When Love Takes Over: Boosting Love Towards Airbnb Brand

**DOI:** 10.1057/s41299-022-00153-9

**Published:** 2022-10-19

**Authors:** Pantea Foroudi, Maria Palazzo, Karanikosova Sabina

**Affiliations:** 1 Middlesex University London, London, UK; 2 Dipartimento Scienze Politiche e Della Comunicazione, University of Salerno, Fisciano, Italy

**Keywords:** Sharing economy, Relationship theory, Airbnb, Brand love, Local community, Prague

## Abstract

The COVID-19 epidemic affected all industries, but the hospitality sector was hardest hit as governments used social distancing to prevent outbreaks. Based on the insights of relationship theory, before social distancing became an essential behaviour spread globally, this study attempts to understand what factors influence love for Airbnb brand, how they are linked together, and how love for Airbnb brand could be beneficial to local business/market. This research focussed on 9 in-depth interviews with Airbnb hosts and 3 focus groups with 13 experts from short-term rentals companies in Prague (22 participants). The outcome of this study provides insights on love towards Airbnb brand, and based on this, it set a start point for deeper, beneficial connectivity between Airbnb users and local community. This research contributes to the expansion of literature about love towards Airbnb brand, its dimensions, and its main outcome. This study suggests that brand identity, communication and branding activities, service attractiveness, guest experience, perceived cultural value, satisfaction and reputation are dimensions of love towards Airbnb brand and have as main outcome love towards local community.

## Introduction

The pandemic crisis and global recession is still widely affecting hospitality and tourism sector (Foroudi et al. 2021). The COVID-19 was spread all round the world by tourists who returned to home countries without being aware of the fact that they had been in infected geographic areas: this was the reason why governments promoted social distancing as a way to keep citizens safe from the virus. After this challenging circumstance and considering its devastating impact on all business industries, especially on hospitality, it is essential to analyse how the pandemic crisis will internationally challenge hospitality and tourism in future and how was the situation in this sector before the crisis started in some specific countries. Thus, reflecting on the rising importance of the topic, researchers and scholars have to try to answer to questions such as is it still worth (and safe) to rent a standard traditional tourism accommodation, or is it better to stay in the house of a host enjoying the real life style of a country?

Many studies until now investigate this issue (Brochado et al. 2017; Akarsu et al. 2020).

The phenomenon of individuals renting lodging to visitors has been reinvented thanks to Internet and is growing significantly thanks to virtual markets where communication and reputation are co-created by hosts and guests at the same time (Liu and Mattila 2017; Xie and Mao 2017).

Airbnb and peer-to-peer short-term rental organisations (e.g. HomeAway, Wimdu) are part of the sharing economy that foster “collaborative consumption” (Ert et al. 2016). The sharing economy is developed with Internet and social networks, and it leverages on consumers having access to goods/services without owning them, and individuals renting out or proposing to others their belongings (Wachsmuth and Weisler 2018). The current economic crisis helped boost sharing economy, but it also gave to this new trend the strength to reinvent different ‘social aspects’ that are at the bases of concepts like branding, communication management and corporate reputation (Mauri et al. 2018; Tussyadiah 2016).

In previous studies, ‘social’ has been identified as an essential aspect of the sharing economy, encompassing consumers’ social relationships (Altinay and Taheri 2019; Cheng 2016). It has also been suggested that the desire for social interaction is often the main driving force behind the phenomenon’s development in the practitioner literature (Lee et al. 2015).

Scholars have given greater attention to peer-to-peer accommodation’s social aspects (Liu and Mattila 2017), but a critical examination is needed in order to better understand how these social factors—deeply embedded in various management and marketing areas—are evolving in the hospitality sector (Dogru et al. 2020; Heo et al. 2019; Varma et al. 2016). Specifically, social aspects can offer significant insights into innovative peer-to-peer accommodation by understanding the role they play as a peculiar element of brand management.

Actually, the rise of Airbnb and peer-to-peer rental services, strongly based on social interactions between hosts and guests, can be considered an innovation within these fields completely renovated by sharing economy (Zervas et al. 2017). With Airbnb’s rapid expansion, the issue of its brand becomes even more decisive (Tussyadiah and Park 2018). Currently, there are several studies that have examined factors affecting Airbnb brand features (Lalicic and Weismayer 2017).

In this context, the Airbnb brand has been extensively analysed (Mody et al. 2019), but there are no studies specifically examining brand love: a concept that can be considered the maximum expression of ‘social interaction’ between hosts and guests.

Although there have been plenty of quite different research regarding brand love and its consequences and antecedents in a sharing-economy context, there have not been many papers focussed on brand love and its results in the peer-to-peer tourism context (Foroudi et al. 2019b; Akarsu et al. 2020; Young and Corsun 2021).

Brand love is a recently emerged concept in tourism’s academic literature (Bairrada et al. 2018). Researchers have revealed that brand love is an antecedent of brand loyalty and co-creation behaviour (Aro et al. 2018; Giovanis and Athanasopoulou 2018). Moreover, scholars have said that brand love is certainly related to willingness to purchase (Liu 2018).

Taking into consideration the insights of relationship theory (Fournier, 1998), this study draws upon the approach of love (Langner et al. 2015) aiming to achieve a set of objectives: (i) It explores in detail previous literature to assess the influence of love towards Airbnb brand and to provide a wider knowledge about Airbnb; (ii) It identifies dimensions that shape love towards Airbnb brand and that are most likely to have a key influence; (iii) It develops a conceptual framework concerning the relationships between love towards Airbnb brand and its dimensions and (iv) it assesses the influence of Airbnb love in Prague.

The remainder of the paper is as follows. In the next section, the study proposes an overview of the main topics regarding Airbnb brand. Afterwards, methods, data selection and research design are detailed. In subsequent section, the results of research are presented. The study ends with implications, conclusions, limitations and suggestions for future research.

## Theoretical Background and Propositions Development

The COVID-19 epidemic, as in the past the Spanish Flu (1918) and the SARS (2003), caused and is still causing huge problems in the global economy, particularly in the tourism sector (Foroudi et al. 2021). In 2019, firstly the Chinese government declared to the World Health Organisation (WHO) that an epidemic of pneumonia was spread in Wuhan due to an unknown virus. Subsequently, in early 2020, the WHO called the virus as new coronavirus. Since then, the COVID-19 cases augmented day by day, causing an enormous economic anxiety which severely impacted on the hospitality sector (Gossling et al. 2020).

The recent circumstances due to the pandemic crisis is still indefinite and full of uncertainty. Nevertheless, it seems that the current situation does not reflect the past outbreak crisis, and it will set the basis for a revolution in the hospitality sector (Foroudi et al. 2021). Therefore, the implication of COVID-19 disaster is unavoidable on users and local business/market.

In attempting to investigate love towards Airbnb brand and its beneficial impact for users and for local business/market, the paper focuses on several issues following the relationship theory. These concepts will be analysed in the next paragraphs.

### Brand Identity

Brand identity is perceived as a unique set of brand associations that a brand, through its strategies, aspires to create, maintain and establish (Foroudi et al. 2019b). Brand identity can be also considered as a combination of sensory components (visual, auditory, etc.) that sustain brand personality (Woods 2020), cause brand recognition, represent brand promise (de Chernatony and Christodoulides 2004) and provide brand distinctiveness (useful in brand positioning) (Worlu et al. 2015). The identity of a brand is conveyed via contact points between company and consumers (Orazi et al. 2017). Analysing Airbnb, the paper will focus on personality, positioning and promise.

*Personality (what makes an Airbnb Super/Host)—*Several research point out that hotels are able to ask for premium price because they set their personality on offering superior services (Jani and Han 2014). Airbnb may not have excellent hosts like hotels but most people only want a place cheaper than the hotel (Airbnb Inc. 2019). According to Statista (2019), 93% of Airbnb users were satisfied with the service they have got, even though the service was not as sophisticated as a five-star hotel would be. Airbnb personality is, in fact, based on the home-service sharing platforms where guests rate homes/services they received.

*Positioning (Airbnb Business Ecosystem)—*Airbnb operates a simple model that benefits stakeholders all round the world (Zervas et al. 2017). It promotes a simple brand positioning that consider the fact that homeowners are attracted to become hosts with their property listed on Airbnb platforms, because it gives them benefits as well as Airbnb managers who receive commissions while acting as brokers between guests and hosts. This means that the host earns good money while sharing his home with guests (Brown 2016).

*Promise—*Airbnb promises to offer lower prices than the conventional hotels (Brown 2016). Today, Airbnb also leverages on premium price as quality standards have raised. In fact, at a lower price, a guest can have an entire home and at the same price they would not be able to secure a hotel room.

The elements of brand identity (personality, positioning and promise), analysed before, affect the brand strategy, which leads to an efficient and synergistic controlled/uncontrolled communication (Kitchen 2017). Thus, it means that brand identity has positive impacts on brand strategy and on controlled/uncontrolled communication (Foroudi et al. 2019b; Melewar et al. 2017). For this reason, the study proposes that

#### Proposition 1

Brand Identity (based on personality, positioning and promise) is expressed through (i) controlled communication (Signature of the platform; Website; Promotion) and (ii) Semi/Uncontrolled communication (word-of-mouth; social media marketing).

### Communication and Branding

According to Leventhal (1996), branding can boost consumer experience and include assets to deliver and communicate the service/product. Companies pursue a wide range of branding models with the aim to build a long-term and sustainable relationship with their consumers leveraging on communication (Strebinger 2014). Controlled and uncontrolled communication is, in fact, the base for demonstrating brand identity and is also recognised as identity-creation process (Schultz and Kitchen 2004). The controlled communication is synergistic and usually more positive, while the uncontrolled communication is more effective in the creation of customer brand loyalty (Melewar et al. 2017). Controlled communication is expressed by signature of the platform, website and promotion, while semi/uncontrolled communication is based on word-of-mouth and social media marketing.

#### Controlled Communication

*Signature of the platform—*Communication is the transfer of information through a medium and the purpose of it, in this case, is to increase awareness of Airbnb (Chaffey and Ellis-Chadwick 2019). The signature of the platform will be the best product and service produced by the organization that competition does not deliver to the consumers. Airbnb will be identified by its ability to serve consumers of all types and levels of disposable income (Inside Airbnb 2019). Services on the Airbnb rooms will be highly contingent to the kind of host on a property but service will be regulated through the home-service sharing platform (Brown 2016). This platform allows guests to rate a property based on the services they received from the host (Kannan 2017).

*Website—*The website is the medium where guests and hosts meet. Airbnb creates the website which allows homeowners to list properties and guests to find a home that attracts them (Wang and Jeong 2018; Chaffey and Ellis-Chadwick 2019). Moreover, accommodation websites, such as Airbnb, have positive responses from engaged guests in sharing content, increasing customers’ loyalty, shaping a positive relationship with a brand and encouraging guests to book their stay (Foroudi et al. 2019b).

*Promotion—*Promotion denotes the attempts to inform, persuade and remind consumers to consider their products over those of competitors (Kitchen 2017). Several promotional strategies can be employed (Gillespie 2015). In fact, using traditional media such as television, radio and print media, the awareness of Airbnb is set to reach the elderly. On the other hand, millennials are reached through the internet and online adverts on billboards.

#### Semi/Uncontrolled Communication

*Word-of-mouth—*Marketing efforts have changed since online and social media gained strong influence and popularity in the market (Goodman 2019). Marketers consider the spreading of positive word-of-mouth and consumer’s recommendation important to create awareness and increase purchases (Leong et al. 2019). The key to reach a positive word-of-mouth is customer satisfaction (Goodman 2019). According to Statista (2019), Airbnb recorded a 93% customer satisfaction rate in 2018 (Airbnb Inc. 2019). Happy customers convey their happiness to people close to them such as friends and relatives (Moise et al. 2019). However, this works also vice-versa: when customers are not satisfied they say it not only to those close to them but also to strangers and this affects the corporate reputation (Filieri et al. 2015).

*Social media marketing—*Social media discussions leads to increase brand awareness, creating brand identity and positive brand associations (Tuten and Solomon 2017). In addition, social media enable companies to project brand image and provide a perfect ‘virtual’ place to share content with readers (Ashley and Tuten 2015). Social media platforms also allow organisations to check competitors, as through social media platforms valuable data from competitors can be collected and be used for boosting the decision-making process (Tuten and Solomon 2017). 60% of Airbnb guests are millennials and social media effectively reaches this target as they and spend a lot of time social platforms (Airbnb Inc. 2019). Thus as discussed above, the study proposes that

##### Proposition 2

Controlled communication and semi/Uncontrolled communication affect guest experience

### The Moderating Role of Service Attractiveness

Li and Jarinto (2012) state that service quality is a significant determinant of customer satisfaction and loyalty in the hospitality industry. The service quality has been also described as composed by several items (Kandampully and Butler 2001). These dimensions are tangibles issues (physical facilities of the property, equipment, etc.), reliability (ability to perform promised services dependably and precisely), responsiveness (willingness to help customers anytime and offering prompt service), assurance (knowledge and politeness of employees and their ability to convey trust and confidence) and lastly empathy (caring, personalized approach and attention offered to customers) (Oakland 2005). These elements will be synthesised in the following sections.

#### Responsiveness

The current Airbnb offering is attractive to both brokers, guests, and hosts and this is because it results in a win–win situation (Brown 2016). With Airbnb, the design, ambience, and type of property all depend on the owner and since there are many owners, customers have a variety of choices to choose from which leads to customer satisfaction (Foroudi et al. 2019a). Airbnb brokers do not have to do anything to the property because they do not own them (Inside Airbnb 2019). Hosts are happy because they get revenue from their property as they get to experience sharing their spaces with people from all walks of life (Statista 2019).

#### Reliability

For Airbnb, like any other business, risks exist (Malazizi et al. 2018). In fact, guests are more likely to go to hosts that have been recommended by other people and this reduces the chances of scammers. In most cases, guests are also urged to make payment once they have seen what they are being offered. With all these precautions, Airbnb has been made more reliable. Besides, hosts have worries that guests they invite to their homes could be dangerous to them. Statista (2019) estimated that 54% of Airbnb guests are female and 46% are male and this is because hosts are sceptical of guests they are going to bring to their homes. Hosts may feel uncomfortable bringing guests of opposite sex to their homes (Malazizi et al. 2018). Nonetheless, Airbnb allows hosts to make their property visible to the gender they want (Brochado et al. 2017).

#### Service Outsourcing (Airport Pickup, Check-In/Out, Customer Care)

Outsourcing is an excellent way of eliminating risks and letting experts do their work (Gonzalez et al. 2011). Airbnb operates a successful model because it does not own any major asset and because it implements outsourcing for many services (i.e. airport pickup, check-in/out, customer care).

*Airport pickup—*Committing to pick guests from the airport will be a lot of work that will require hiring drivers and taxicabs which is not its core business. Airbnb can outsource airport pickup to the businesses that do the pickup as their core business and this allows Airbnb to concentrate on its core businesses and this increases the rate of customer satisfaction (Feng et al. 2019).

*Check-in/out—*Checking in/out is part of the responsibility of the host and statistics show, homes, where the host stays with the guests, bring in much more revenue (Cheng and Jin 2019). During the check-in and check-out process, the host can take details of guests such as emails for future communication which is a strategy to increase additional sales in the future (Foroudi et al. 2019c).

*Customer care—*Customer care is the process of looking after customers to guarantee their satisfaction and interaction with brand and services (Foroudi et al. 2019b). Customer service should also not be outsourced because the host is in a better position to cater to needs of customers (Gonzalez et al. 2011). Customer care and the process of check-in/out are closely linked (Reid and Bojanic 2009).

#### Dining Options (In-House, Outsourced, Affiliate Programme)

*In-house—*The host will take care of guests by cooking for them. Guests are also free to prepare their desired meals and if it is not available they can order. This flexibility is what makes Airbnb experience far much better than hotels (Cheng and Jin 2019).

*Outsourced—*On some occasions, guests can be numerous maybe because hosts run a business and therefore they manage many properties. When the number is high, this may require the services of a professional to prepare breakfasts. In this case, outsourcing the cooking services to a professional is the best thing to do to ensure that guests receive breakfasts (Cheng and Jin 2019).

*Affiliate Programme—*Airbnb solely runs bed & breakfast programmes alone and that is its core business. If there are other affiliated programmes, guests will have to pay for them separately (Brown 2016). Thanks to its partners, Airbnb is able to offer programmes for tourists: partnering with local organisations ensures that each business concentrates on its core business but at the same time offer guests an excellent experience (Cheng and Jin 2019).

#### Guest Personalization (Chat-Bots, Welcome Letter, House Rules, City Guides)

*Chat-bots*—Chat-bots are computer programmes designed to simulate conversation with human users, and they are a good way to respond to routine questions (i.e. sizes of rooms, price range) (Følstad and Brandtzæg 2017). Chat-bots ensure that clients’ queries are attended in time and even if the issue is not solved, it leaves clients knowing that the query is being attended by service provider (Følstad and Brandtzæg 2017).

*Welcome letter—*Letters are personalized and they make guests/clients feel special. When hosts take time to write letters as a sign of gratitude for guests, it makes them want to come back again. A welcome letter is equally effective as it serves the purpose of introducing host to guests, services offered and affiliated programmes (Camilleri 2018).

*House rules—*House rules (i.e. guide book, guest book) are contingent to the host. These rules are availed on website as terms and conditions which guest has to agree before proceeding to rent rooms. In some instances, during check-in process, a template with house rules is provided (Inside Airbnb 2019).

*City guides—*As mentioned earlier, Airbnb only provides guests with bed & breakfast and affiliated programmes are outsourced to other organisations. City guides, for example, are people who take tourists around the city and inform them about structures and history. City Tourism offers these services which means Airbnb does not have to offer them (Inside Airbnb 2019). Finally, as discussed above, the study proposes that

##### Proposition 3

Service attractiveness reinforces the relation between communication and guest experience

### Guest Experience

Experience leverages on memorable events that engage individuals and is ascertained that guest’s emotional and spiritual engagement relate on experience (Gilmore and Pine 2007). Creating excellent guest experiences has become a main objective in achieving competitive advantages and developing guest’s loyalty in the hospitality industry (Gentile et al. 2007). Based on Tynan and McKechnie (2009), a great guest’s experience can be achieved by creating enjoyment, entertainment, and uniqueness (Hyde and Harman 2011). Nevertheless, it should be taken into account that guests will evaluate their experience depending on their own expectations, feelings, the overall context and spent moments (Holbrook 2006). Every Airbnb host desires to give guests a new experience that will result in loyalty. Airbnb hosts are encouraged to engage with guests even before they arrive and this can be done through chat-bots and customer care representatives (Følstad and Brandtzæg 2017). Thus considering, that experience can have a significant influence on guests’ satisfaction with a received service (Wu et al. 2018), the study proposes that

#### Proposition 4

Positive guest experience positively affects satisfaction

#### Proposition 5

Positive guest experience positively affects perceived cultural value

### Perceived Cultural Value

Definition of a cultural values refers to the culture’s mind-set and the understanding shared by members of a society: it creates a code of conduct that affect attitudes, opinions and behaviours of a certain society (Kluckhohn 1951). The perceived value is a construct linked with quality, economic, emotional, social, novelty, and knowledge dimensions (Oliver 2014). It was disclosed that perceived value influences satisfaction in a positive way (Prebensen and Xie 2017). Prahalad and Ramaswamy (2004) discussed that the meaning of perceived value has been changing: basically, it shifts the perspective from a product-centric financial perspective to a more experience-oriented perspective. In term of perceived value, the mission of Airbnb is to create a world where anyone can belong anywhere. Thus, it tries to reach the purpose with over 150 million users worldwide and in over 191 countries (Airbnb Inc. 2019). From consumer point of view, culture created by Airbnb is a value: together host and guest can interact and other than sharing the space, they can share experiences, knowledge and authentic culture (Brochado et al. 2017). This is something unique that customers cannot get from hotels and is one of the reasons why people are shifting their focus towards Airbnb. Moreover, Airbnb is a symbol of collaborative lifestyle (Brown 2016). Therefore, the study proposes that

#### Proposition 6

Perceived cultural value affects satisfaction

### Satisfaction

Customer satisfaction is defined as a business philosophy that relies on the need of creating value for customers and fulfilment for customer’s expectations (Dominici and Guzzo 2010). Different organisations in a competitive market such as peer-to-peer accommodation platforms are aiming at reaching customer satisfaction (Tussyadiah 2016). To improve guest satisfaction, Airbnb employs personalised marketing which is a strategy founded on the delivery of promotional content to users on a more individual basis. This is achieved through digital data collection (Liu and Mattila 2017). All information is collected and promotional content is set on guests’ searches as personalized promotion ensures that a guest is connected to the right host based on the guest’s preferences (Mody et al. 2019). Satisfaction can become brand love. According to Carroll and Ahuvia (2006), brand love is an emotional feeling expressed by customers towards brands. Based on their definitions, Schnebelen and Bruhn (2018) consider satisfaction as an antecedent of brand love and stated that love is “experienced by some, but not all, satisfied consumers” (p. 81). Other authors state that being satisfied with a brand for a period of time can make individuals love brands (David 2018). In addition, Whang et al. (2004) highlighted that in order to reach love for a brand, clients need to be first satisfied of brands. Therefore, the study proposes that

#### Proposition 7

Satisfaction affects love towards brand

### Love Towards Brand and Towards Local Community

Carroll and Ahuvia (2006) defines brand love as the degree of emotional attachment satisfied consumers have for a brand. Love is linked to positive feelings and is a consequence of positive brand relationship (Fehr and Russel 1991; Albert et al. 2008). Based on Keh et al. (2007), brand love is not just about passionate emotions but it also includes a long-term brand commitment and brand loyalty (Kang 2015). Batra et al. (2012) say that love towards brand is a crucial factor which provides stability to brand in a changing world. Love towards the Airbnb brand can be proven by numbers. First and foremost, Airbnb offers listings in over 191 countries (Statista 2019) and has over 150 million users. Moreover, Airbnb (2019) observes that tourists do not just desire to rent a room, nonetheless also pursue to co-create their travel experiences (Liu and Mattila 2017). They travel to different destinations and select properties that involve them on a personal level. Airbnb kindles the communal feeling of sharing, ‘what is mine is yours’. This is a culture that has been brought about by the economy of sharing (Zervas et al. 2017). Human beings need each other to have the best experience in life and to have a wide experience of the host and local community. Nevertheless, not much is known about the processes in play when customers co-create value and feel brand love through shared experience (Brochado et al. 2017). In light of the above, the formulation of the propose included in the model appears evident.

#### Proposition 8

Love towards brand increases love towards local community

### The Moderating Role of Reputation

Corporate reputation is a mental picture of an organisation based on aggregated multiple images held by internal and external stakeholders (Fombrun and Van Riel 1997). Shareholders before investing in a company rely on reputation when making investment decisions (Dowling 1986). A company’s uniqueness improves its visibility, and positively impacts the public impression (Fombrun and Van Riel 1997). For this reason, managers should be concerned about reputation including Airbnb hosts (Ert et al. 2016). Considering Airbnb, prior studies have identified that reputation is able to affect many issues in isolation; however, there is no research that discusses the operationalisation of variables related to reputation, brand love and satisfaction. So, taking into account past studies, the study propositions that

#### Proposition 9

Reputation reinforces the relation between satisfaction and love towards brand

## Design and Methodology

The research methodology employs two main research methods (in-depth interviews and focus groups). These methods were used to investigate thematic areas of this research which take account of Airbnb identity—Users’ perception, communication and branding, service attractiveness, guest experience, perceived cultural value, satisfaction, love towards brand, reputation and love towards community. Qualitative methodology is suitable for this research because it delivers a deeper understanding of consumer’s opinions (Bryman and Bell 2015); it provides an accurate portrayal of consumers’ thoughts, perceptions, interests, lifestyle, behaviour and preferences (Bell 2010). The research data were conducted in August 2019 in Prague, Czech Republic. For the qualitative research, this study has a sample of 22 respondents, who were selected through LinkedIn and Airbnb platforms. The sampling frame consists of 13 respondents (Airbnb Hosts) who attended focus group interviews, and 9 respondents (experts from Prague’s short-term rentals companies, which collaborate with Airbnb, manage and offer properties through Airbnb), who attended in-depth interviews. Out of 22 respondents, 15 were men and 7 were women, that means the majority of respondents were men.

### The Setting: Prague Market (Current Situation and Airbnb Impact in Prague)

The current situation in Prague market is conducive to Airbnb. The number of visitors to the city is increasing every day and is projected to reach 21 million by 2020. Most of these people will be attracted to Airbnb over normal hotels mainly because of the lower prices as they see the value for their money. Currently, Prague accounted for over 18 million overnight stays and hotels accounted for 40% of these stays (Prague City Tourism 2019). Airbnb is taking over the Prague market quickly due to the ease at which people can find accommodation, wide range of rooms giving them choices and affordability (Ert et al. 2016).

### Data Collection

Data were collected using two methods: in-depth analysis and focus groups.

In-depth interviews are a qualitative data collection technique that embraces direct, one on one engagement with the participants (Marshall and Rossman 2011; Bryman and Bell 2015).

Focus groups, on the other hand, refer to a group of experts in a certain field ten or fewer who volunteer to discuss a certain topic, product or idea (Ateljevic et al. 2007). In-depth interviews and group discussions are very advantageous to combine as a valuable resource that conveys a new perspective to existing data (Creswell 2013). The data collected from the interviews and focus groups supplied the information and insights to this research and facilitated to add more data, which was not identified in the literature review. Additionally, the interview protocol (see Table 1) was designed to ensure that all areas of interest were covered. However, all respondents were allowed to freely move from subject to subject without necessarily sticking to the order of questions in the interview protocol. The usage of open-ended questions is essential for an exploratory study like this. Once the data have been collected the researcher summarized and attempted to find meaning from the data. The methods employed by the researcher to analyse and interpret the data included describing the data and identifying themes (Creswell 2014). The collected data were also coded and reduced to a manageable form. This was accomplished by dividing the data into main categories taken from literature review.

**Table 1:** In-depth interviews schedule

Interview date	Interviewee	Profile of participants	Interview approx. duration (min)
In-depth interview schedule			
09.08.2019	Expert 1	Experts from short-term rentals companies which collaborate with Airbnb or manage and offer properties through Airbnb	60
13.08. 2019	Expert 2	Experts from short-term rentals companies which collaborate with Airbnb or manage and offer properties through Airbnb	60
15.08.2019	Expert 3	Experts from short-term rentals companies which collaborate with Airbnb or manage and offer properties through Airbnb	60
19.08.2019	Expert 4	Experts from short-term rentals companies which collaborate with Airbnb or manage and offer properties through Airbnb	60
20.08.2019	Expert 5	Experts from short-term rentals companies which collaborate with Airbnb or manage and offer properties through Airbnb	60
21.08.2019	Expert 6	Experts from short-term rentals companies which collaborate with Airbnb or manage and offer properties through Airbnb	60
21.08.2019	Expert 7	Experts from short-term rentals companies which collaborate with Airbnb or manage and offer properties through Airbnb	60
22.08.2019	Expert 8	Experts from short-term rentals companies which collaborate with Airbnb or manage and offer properties through Airbnb	60
23.08.2019	Expert 9	Experts from short-term rentals companies which collaborate with Airbnb or manage and offer properties through Airbnb	60
Topics discussed			
Airbnb identity, communication and branding, service attractiveness, guest experience, perceived cultural value, satisfaction, love towards brand, reputation and love towards community			

### In-Depth Interviews

In-depth interviews were instrumental in comprehending how Airbnb can stand out in short-term business in Prague. The interviews focussed on understanding whether the selected constructs included in the model have been responsible for the increase in the number of people using Airbnb as an alternative to normal hotels. In-depth interviews were conducted with experts from Prague’s short-term rentals companies, which collaborate with Airbnb or manage and offer properties through Airbnb: LetMeInn, EasyBnB, PRAGUE STAY, SevenKeys, Blahobyty, Empirent Apartments, Honeco, Carebnb, Czech Association of Landlords and Accommodation Providers—ČAPUS. Every interview with each expert covered all constructs: Airbnb identity—Users’ perception, communication and branding, service attractiveness, guest experience, perceived cultural value, satisfaction, love towards brand, reputation and love towards community. The duration of each in-depth interview took approximately 60 min and was recorded and transcribed verbatim to ensure the reliability of the data. The table below shows the schedule of interviews and discussed topics.

### Focus Group

Focus groups were done in three face-to-face interviews, two of them include 5 hosts and one of them 3 hosts. Focus groups provided an opportunity for participants to be in one room and discuss their different perspectives and it also allowed retrieving any information that had been forgotten during the in-depth interviews. Participants of the Focus Group interview—were Airbnb Hosts—who manage more than 2 apartments in Prague and have had more than 3 years’ experience with Airbnb—the experience of being a Host. Every focus group interview covered all constructs. The duration of each focus group interview took approximately 90 min and was recorded and transcribed verbatim to ensure the reliability of the data (see Table 2).

**Table 2:** The focus groups

Interview date	Number of participants	Profile of participants	Interview approx. length (min)
12.08.2019	5 Hosts	Airbnb Hosts—who manage more than 2 apartments and have had more than 3 years	90
14.08.2019	5 Hosts	Airbnb Hosts—who manage more than 2 apartments and have had more than 3 years	90
16.08.2019	3 Hosts	Airbnb Hosts—who manage more than 2 apartments and have had more than 3 years	90
Focus groups’ topics			
Airbnb identity, communication and branding, service attractiveness, guest experience, perceived cultural value, satisfaction, love towards brand, reputation and love towards community			

## Findings and Discussion

In this section, results are explained to establish and support the development of every construct of this study. The content analysis of this research has identified several influencing factors of the love towards the brand and community. In addition, results of the in-depth interviews are presented alongside with the focus group interviews.

### Brand Identity

#### Perception and Identification with the Brand Personality

Perception indicates how consumers perceive Airbnb brand. The brand of a commodity affects the willingness of consumers to spend money on the product/service (Pappu et al. 2005). Findings from the qualitative research pointed out that brand perception is key to the success of Airbnb. Airbnb has no physical features to make it unique but what makes it different is the position Airbnb occupies in the minds of its consumers. As results suggest, interviewees used positive words to express their perception of the brand. Four of the interviewees from in-depth interviews recognized Airbnb as a brand that allows people regardless of their level of income. All three focus groups agreed that Airbnb is a form of sharing economy that allows anyone to belong anywhere and get to have a better tourist experience. Moreover, interviewees identified Airbnb personality with travelling: anytime they thought of travelling, they thought of Airbnb. Perception and thoughts have a significant impact on a brand (Pappu et al. 2005). The following quotes from the interviewees express their identification with the brand;

I like to travel via Airbnb, so for me and my identification with Airbnb is traveling and connectivity with foreign people. (Interview Expert 1)

This identification confirms Airbnb (2019) observation that tourists pursue to co-create their travel experiences.

#### Reason for Believing in Brand Promise

The results of the study indicate that managers accept Airbnb because of its simple yet effective business model and original promise. Managers claim that platform is easy to use and allows both parties to be in communication, unlike other platforms such as the booking.com. She stated

Moreover, a commission on Airbnb is the lowest. Airbnb is intuitive, easy to manage, while Booking.com is more complex (Interview Expert 5).

Because of the easy business model and promise, it is very convenient to both host and guest as there are no complications. Another of the interviewees said “Because Airbnb does not mean just a financial reward, but it is a personal reward too for hosts and guests alike” (Interview Expert 2). This shows that Airbnb is conveying more value to both guests and hosts, and this explains the gradual growth of the organization.

#### Evaluation of the Situation in Prague: Positioning Airbnb in Prague

The Airbnb business model is simple as it was designed to make it easier for new entrants to join the platform and help the Airbnb concept expand quickly. The model has achieved its purpose as the growth of Airbnb has been exponential, and respondents agree that competition is high in Prague. The dangers of exponential growth are also being experienced, and one of them is a reduction in the quality of service offerings. A participant commented

The number of apartments is growing, and Airbnb is losing its identity. It is just another accommodation option. (Interview Expert 2)

With growth, more hosts are offering Airbnb services, yet the number of guests is not growing at the same rate. This is why in the beginning, Airbnb was profitable to hosts, but at the moment, the earnings are not so high because the market is oversized. For guests, it is good news because they have an array of options to choose from, but for the host, they have to attract guests, as participant’s comment,

There is a very high competition in Prague. Primarily demanded apartments are with a very good location, in the centre of Prague or apartments that are in some way exceptional. (Interview Host 11)

### Communication & Branding

#### Controlled Communication

Results show that communication is set in Airbnb platform as this is where guests meet their hosts for the first time. Communication can go on in this platform, and follow-ups can be done through email. Emails appear to be commonly used as it allows the host to show their apartments by sending pictures or information in PDF which will make the guest more attracted to homes. Follow-up can also be done on the Airbnb chat window, but the downside is that it does not allow the attachment of files as explained by expert 4 in the interview.

#### Uncontrolled Communication (Word-of-Mouth, Social Media Marketing)

Most communication is through the controlled channels, but hosts have no control over what guests will say about their apartments and services on social media. Hosts have pages on social media platforms such as Facebook and Instagram, where they showcase their property and provide links to drive guests to their Airbnb platforms for booking. A participant commented on the following:

We have a profile on Facebook and Instagram, where we are active. In our company, we have a team of people who are the focus on social media and online promotion/marketing. (Interview Expert 7)

On the other hand, typical Airbnb Hosts, who are not belonging to any short-term rentals company, are not active on social media channels. Mostly they do not even have a profile on social media platforms because they have not found it useful. Two of the interviewees quoted it as follows:

We gave a chance to Instagram profile, but we have not found out beneficial yet and I think we will not find out in the future. Our target is not on social media platforms, so right now we do not concentrate on this. (Interview Host 8)

We used to have a profile on Instagram and Facebook, but we did not find it profitable for us in any cases. In terms of our website, we have not decided yet. (Interview Host 13)

Uncontrolled communication is essential as controlled communication. This is how hosts get guests to visit their profiles on the Airbnb platforms. Guests who are satisfied express their gratitude on the Airbnb platform as well as social media. The statements read as follows:

It works a lot, we have many clients because of this. Recommendation is the best promotion. (Interview Expert 4)

Yes, it essential this kind of promotion. It brings new clients and as well as new incoming guests. We are glad that this works very well for us. (Interview Expert 2)

Sometimes we have guests who come directly based on the recommendation, so for us, it’s a very good advertisement. (Interview Host 7)

### Service Attractiveness

#### Attractiveness of Properties in Terms of Responsiveness and Reliability

Results show that the style of the apartment and the equipment can make up for not being in a good location. With increased competition, to keep guests coming, apartments now have to convey something unique to guests in terms of responsiveness and reliability. One respondent said

Our terraces with (360°) view of Prague. On 100%. We have large terraces in each apartment with an amazing view. But we also give total attention to all our guests needs and try to be as flexible as possible with them (Interview Host 2).

#### Service Outsourcing

Service outsourcing exhibited a mixed outcome from people who were interviewed. It revealed those hosts with more guests outsource services such as laundry, decoration, cleaning, and breakfast, on the other hand, those with fewer guests do everything themselves. The host receives guests from different parts of the world, and unlike hotels, it is hard to determine what guests will enjoy for breakfast. While some hosts like to play it safe by not offering breakfast and in its place, they provide juice, beverages, and snacks others go to the extent asking guests’ preferences for breakfast before they visit, and the host would make the breakfast available through outsourcing.

#### Dining Options (In-house, Outsourced, Affiliate Programme)

Airbnb no longer provides dining, and this is because Airbnb is solely to provide a place for guests to spend a night. Other services are add-ons to increase value and improve customer loyalty. Hosts also do not collaborate with restaurants to offer dining options.

Participants commented

I do not serve breakfast, but I recommend a lot. Guests usually want to recommend restaurants with cheap prices, yet quality food. Anyway, based on my experiences – local bistros are very favourite. (Interview Host 11)

Neither us. I would love to serve breakfast to guests, but it is time-consuming, we do not have just one apartment. We were thinking about outsourcing breakfast…However, in the meantime a lot of cafes (with a wide range of breakfasts) have spread around Prague, so we gave up on outsourced breakfast. In our Guide Book, there is a list of recommendations with these cafes around the apartment. (Interview Host 13)

No, we don’t offer breakfast. In the Guest Book, we have a list of recommended cafes (which we have tested on our own) in the area of the apartment. The cafes are open every day in the morning and offer a wide range of breakfasts. Guests are very always satisfied and they enjoy visiting the local bistros and meeting local people. We do not recommend food delivery apps, we rather send our guests outside to enjoy and experience Prague’s life.

(Interview Expert 6)

No, we do not serve breakfast. It is a pity that this original idea of Airbnb has almost disappeared, but nowadays Airbnb is more business than “couch-surfing”. People can have breakfast anywhere near the apartment. There are plenty of cafes, bistros, and restaurants in Prague offering all types of breakfast. (Interview Expert 4)

We don’t have affiliate programmes. Rather, we do not seek these collaborations. Once you send guests somewhere, you would have to guarantee the quality, which is never certain. We do not want unnecessary complications. (Interview Expert 2)

The following comment reinforces the initial statement made by Expert 2 when asked whether they would recommend sending their guests to restaurants and earn commissions; “No, and we do not like this idea and this kind of thinking—to gain profit from everyone and everywhere, that is not our vision, and we do not want to be seen in this way.” (Interview Expert 1).

#### Guest Personalization

Results showed that when hosts have many guests they do not personalize services to suit their guests. One interviewee said

Our business is too big to concentrate on such small things (Interview Expert 2).

This suggests that hosts with fewer guests could personalize their service offering to meet the needs of the few guests and they are more likely to do that.

### Guest Experience

Results depict that Airbnb host receives good reviews most of the time. Respondents said that they use guestbook where guests leave their contacts and review of services. Responding to reviews as quickly as possible is also part of the guest experience. Reading the reviews, hosts know how to improve their services to meet the desire of guests. Others use the phrase, “the customer is always right”, and hence listening to them improves the guest experience.

### Perceived Cultural Value

The culture of guests does not affect the service offering of Airbnb. The results indicate the hosts do not have to consider the guest’s lifestyle and furnish homes to suit their culture.

For example, two of the interviewees confirmed it as follows:

No, in our case this is inconceivable. (Interview Expert 6)

No. It would be certainly operationally exacting. (Interview Expert 7)

Some hosts also admitted that some guests are demanding, but if they are paying extra money for the additional services the host will provide. The language barrier is no longer a huge problem, and this is because most people can speak English and those who cannot have an array of translating applications that can be used in smartphone. Guests, on the other hand, enjoy the local culture and is the reason why they choose Airbnb over other options. Airbnb allows guests to interact with locals, get to experience their ways of life, and appreciate other cultures. The statement reads as follow:

I give people a personalized view, local tips and this is exactly, what they cannot get from hotels. I call it the added value of Airbnb. (Interview Host 12)

### Satisfaction, Reputation, Love Towards Brand and Towards Community

Results show that guests enjoy constant communication. Guests rarely return, and respondents believe Prague is a small city and not the most attractive in Europe. Value for the customer is created through communication and integrity as customers appreciate hosts who are truthful about their apartments. In general, the service experience (in this case the entire guest stay in Airbnb apartment) can have a significant influence on overall guests’ satisfaction (Wu et al. 2018). Satisfaction, as said before, can be considered the basis of brand love.

**Fig. 1:**
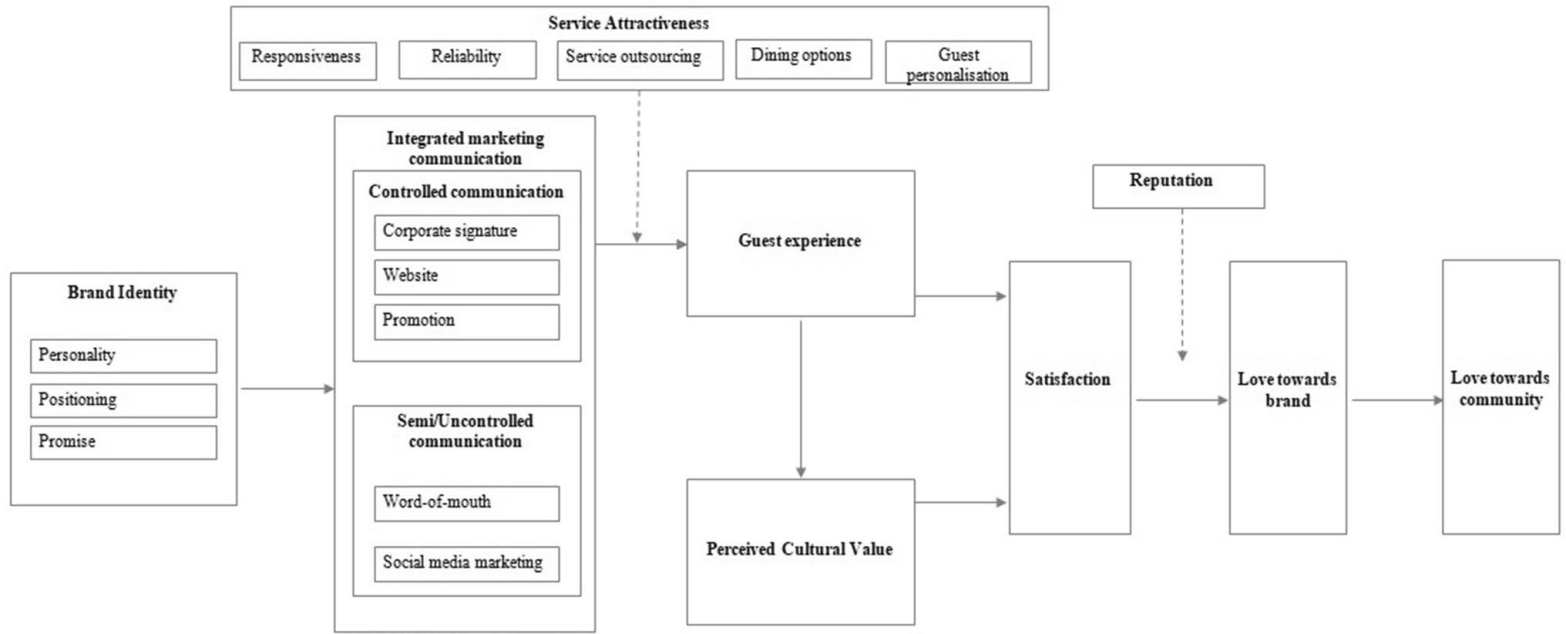
The research conceptual model

Carroll and Ahuvia (2006) clarified the brand love is a feeling that satisfied consumer has towards a particular brand and expresses the degree of passionate emotional attachment. According to the theoretical and empirical findings, it is important to provide the best Airbnb service to guests and create the best possible stay for guests to make them feel satisfied, grateful and in love with the Airbnb brand and connected with the local community. After all, guests are going to feel satisfaction and love towards the Airbnb brand and the local community. Further, this emotional attachment will be as well profitable for the local community. Based on the literature review and on results of the qualitative study, we developed a framework (Fig. 1).

## Theoretical and Practical Implications

Airbnb users’ thoughts and feelings about the Airbnb brand were summarised in the conceptual framework. The model suggests that it is necessary to evocate in users the following responses: positive feeling towards Airbnb identity and its core values/mission/visions, a sympathy with Airbnb branding and communication, satisfaction with provided service and experiences, and pleasure to work in multicultural environments. The result of fulfilling these requirements can lead to a positive reputation and good feeling towards Airbnb brand: the love towards Airbnb brand. Because Airbnb brand is connected with locals, this love towards Airbnb brand will be also transformed into an emotional attachment to local community. This study also proved that each dimension of love towards Airbnb brand has a meaningful connection with the outcome proposed in the conceptual framework. In other words, in case of failure of dimensions of love towards Airbnb brand, there will be just a lack of positive feelings towards the Airbnb brand and lack of benefits for the Airbnb users and local community.

All findings obtained in this research have important managerial implications. Czech users love the core idea of Airbnb brand, they perceived themselves to be part of Airbnb community thus they displayed mainly positive feelings towards Airbnb identity.

Talking about communication and branding, Airbnb users prefer online communication. Moreover, according to this study, the service offer is quite extensive in Prague but guests have a wide choice of possible services, some of them can already be compared with the hotel services. In terms of guests’ service attractiveness, the offer can be enriched thanks to local organisations’ help able to set a potential mutual cooperation, including benefits not achievable through affiliate programme.

It is already well-known that Airbnb users bring economic benefits to the locals. For this reason, the research suggests an implementation of wider cooperation with the community. In other words, it involves coming back to the true meaning of the brand “Airbed and Breakfast” with an experience set on locals service. Besides all benefits reachable for incoming guests, this deeper cooperation would be also beneficial for hosts and local businesses.

Ultimately, the profit may be reflected in the Prague’s growing economy and may promote a healthier development of the sharing economy in Prague. In terms of cultural values, the research shows that guests’ culture has a small effect on the evaluation of apartments. In case that Airbnb host provided everything promised, the consequence of that is an overall positive guest experience. That leads to guest satisfaction, and positive reputation, which is the main condition and the starting point of love towards Airbnb brand. Moreover, thanks to Airbnb guests’ open attitude, it would be much easier to also develop love towards local community.

## Conclusions, Limitations and Future Research

The global pandemic disruption and international economic crisis, particularly in hospitality sector, entails a dynamic transition and a fast-moving adoption of the strategy of the “New Normal” (Gössling et al. 2020). Trying to set the basis for the “New Normal”, this research contributes to the expansion of literature about the love towards the Airbnb brand, its dimensions, and its main outcome. Firstly, developing a conceptual framework based on data from previous studies and secondly, testing it in Prague short-term rental industry, this study suggests that brand identity, communication and branding, service attractiveness, guest experience, perceived cultural value, satisfaction and reputation are dimensions of the love towards Airbnb brand and that the main outcome is the love towards the local community.

The paper shows several limitations and offers some insights for future research. The data were collected at a particular point in time, future exploration can focus on longitudinal research and boost the conceptual framework and research approach in order to examine how love towards Airbnb is perceived before, during and after the pandemic crisis.

Besides, the paper focussed on one specific geographic area, and further study can take into account different cities and compare the findings with the achieved results to attain wider level of generalization.

Furthermore, culture in different nations may involve different levels of engagement with the Airbnb brand. This could be measured by further researchers, collecting cross cultural data, in order to achieve further generalizability.

Due to the significance of the issue, the peculiar features of the topic and time limitations, qualitative methods were selected to collect the data. However, further researchers may assess the strength of the proposed framework employing other methods, in which results will be triangulated.

Finally, it could be useful to collect data from developing geographic areas which have a different approach to hospitality. It may enhance the comprehension into the proposed framework to compare the developed areas with developing countries.
